# Supplementary material for: Comparative multiomics analysis of cell physiological state after culture in a basket bioreactor
Source: Sci Rep. 2022 Nov 23;12:20161. doi: 10.1038/s41598-022-24687-4 (PMC9686226; doi:10.1038/s41598-022-24687-4)
Supplement: Supplementary file 1 — Supplementary Information 1. [file 41598_2022_24687_MOESM1_ESM.zip › raw data/Transcriptomic raw data/6.Enrichment/2.KEGG/TE1vsTC1/ALL/plot/TE1vsTC1.html]

### TE1vsTC1 KEGG通路富集列表

---

| KEGGID | Description | GeneRatio | BgRatio | pvalue | padj | geneID | geneName | keggID | Count | Up | Up\_Gene\_id | Down | Down\_Gene\_id |
| --- | --- | --- | --- | --- | --- | --- | --- | --- | --- | --- | --- | --- | --- |
| csab04630 | JAK-STAT signaling pathway | 12/175 | 111/5900 | 0.0000972 | 0.0183637 | 103226390/103235305/103237005/103246489/103236305/103218491/103219268/103237430/103247197/103238870/103224900/103223920 | IL6/IL11/IL7/CCND1/IL15/PTPN6/CNTFR/MYC/MCL1/SOCS2/PIK3R3/IL6R | csab:103226390/csab:103235305/csab:103237005/csab:103246489/csab:103236305/csab:103218491/csab:103219268/csab:103237430/csab:103247197/csab:103238870/csab:103224900/csab:103223920 | 12 | 7 | 103226390/103235305/103246489/103236305/103237430/103247197/103238870 | 5 | 103237005/103218491/103219268/103224900/103223920 |
| csab04060 | Cytokine-cytokine receptor interaction | 15/175 | 170/5900 | 0.0001391 | 0.0183637 | 103226390/103235305/103241278/103246725/103237005/103236305/103219268/103235789/103225587/103242551/103242198/103225819/103223920/103221402/103242710 | IL6/IL11/IL1B/BMP2/IL7/IL15/CNTFR/CXCL1/TNFRSF1B/TNFRSF6B/CXCL16/TNFRSF18/IL6R/TNFRSF21/CCL2 | csab:103226390/csab:103235305/csab:103241278/csab:103246725/csab:103237005/csab:103236305/csab:103219268/csab:103235798/csab:103225587/csab:103242551/csab:103242198/csab:103225819/csab:103223920/csab:103221402/csab:103242710 | 15 | 5 | 103226390/103235305/103246725/103236305/103221402 | 10 | 103241278/103237005/103219268/103235789/103225587/103242551/103242198/103225819/103223920/103242710 |
| csab04621 | NOD-like receptor signaling pathway | 12/175 | 131/5900 | 0.0004669 | 0.0393893 | 103226390/103224057/103241278/103224476/103239154/103230198/103235789/103224480/103240296/103215179/103242710/103239156 | IL6/LOC103224057/IL1B/LOC103224476/OAS1/IKBKE/CXCL1/LOC103224480/TNFAIP3/CARD6/CCL2/OAS2 | csab:103226390/csab:103224057/csab:103241278/csab:103224476/csab:103239154/csab:103230198/csab:103235798/csab:103224480/csab:103240296/csab:103215179/csab:103242710/csab:103239156 | 12 | 2 | 103226390/103240296 | 10 | 103224057/103241278/103224476/103239154/103230198/103235789/103224480/103215179/103242710/103239156 |
| csab04668 | TNF signaling pathway | 10/175 | 98/5900 | 0.0005968 | 0.0393893 | 103226390/103241278/103222156/103236305/103235789/103225587/103218917/103240296/103224900/103242710 | IL6/IL1B/EDN1/IL15/CXCL1/TNFRSF1B/TRAF1/TNFAIP3/PIK3R3/CCL2 | csab:103226390/csab:103241278/csab:103222156/csab:103236305/csab:103235798/csab:103225587/csab:103218917/csab:103240296/csab:103224900/csab:103242710 | 10 | 4 | 103226390/103222156/103236305/103240296 | 6 | 103241278/103235789/103225587/103218917/103224900/103242710 |
| csab05162 | Measles | 10/175 | 110/5900 | 0.0014712 | 0.0776812 | 103226390/103241278/103246489/103239154/103230198/103240296/103224900/103223677/103228406/103239156 | IL6/IL1B/CCND1/OAS1/IKBKE/TNFAIP3/PIK3R3/HSPA6/CBLB/OAS2 | csab:103226390/csab:103241278/csab:103246489/csab:103239154/csab:103230198/csab:103240296/csab:103224900/csab:103223677/csab:103228406/csab:103239156 | 10 | 4 | 103226390/103246489/103240296/103228406 | 6 | 103241278/103239154/103230198/103224900/103223677/103239156 |
| csab05171 | Coronavirus disease - COVID-19 | 12/175 | 163/5900 | 0.0031349 | 0.1379335 | 103226390/103241278/103221732/103244654/103221259/103239154/103230198/103224900/103233571/103223920/103242710/103239156 | IL6/IL1B/LOC103221732/HBEGF/RPL22L1/OAS1/IKBKE/PIK3R3/CFD/IL6R/CCL2/OAS2 | csab:103226390/csab:103241278/csab:103221732/csab:103244654/csab:103221259/csab:103239154/csab:103230198/csab:103224900/csab:103233571/csab:103223920/csab:103242710/csab:103239156 | 12 | 3 | 103226390/103244654/103221259 | 9 | 103241278/103221732/103239154/103230198/103224900/103233571/103223920/103242710/103239156 |
| csab04657 | IL-17 signaling pathway | 7/175 | 69/5900 | 0.0041037 | 0.1547682 | 103226390/103241278/103230198/103235789/103240296/103242634/103242710 | IL6/IL1B/IKBKE/CXCL1/TNFAIP3/TRAF4/CCL2 | csab:103226390/csab:103241278/csab:103230198/csab:103235798/csab:103240296/csab:103242634/csab:103242710 | 7 | 3 | 103226390/103240296/103242634 | 4 | 103241278/103230198/103235789/103242710 |
| csab05418 | Fluid shear stress and atherosclerosis | 9/175 | 111/5900 | 0.0054464 | 0.1740865 | 103241278/103243277/103215685/103222156/103220724/103218213/103218702/103224900/103242710 | IL1B/ITGB3/PLAT/EDN1/SDC1/GPC1/MGST1/PIK3R3/CCL2 | csab:103241278/csab:103243277/csab:103215685/csab:103222156/csab:103220724/csab:103218213/csab:103218702/csab:103224900/csab:103242710 | 9 | 3 | 103243277/103222156/103218702 | 6 | 103241278/103215685/103220724/103218213/103224900/103242710 |
| csab04640 | Hematopoietic cell lineage | 6/175 | 56/5900 | 0.0059348 | 0.1740865 | 103226390/103235305/103241278/103243277/103237005/103223920 | IL6/IL11/IL1B/ITGB3/IL7/IL6R | csab:103226390/csab:103235305/csab:103241278/csab:103243277/csab:103237005/csab:103223920 | 6 | 3 | 103226390/103235305/103243277 | 3 | 103241278/103237005/103223920 |
| csab00590 | Arachidonic acid metabolism | 5/175 | 43/5900 | 0.0083793 | 0.2031011 | 103235528/103237812/103245108/103218549/103220573 | PLA2G4C/LOC103237812/LTC4S/CBR3/PLB1 | csab:103235528/csab:103237812/csab:103245108/csab:103218549/csab:103220573 | 5 | 1 | 103237812 | 4 | 103235528/103245108/103218549/103220573 |
| csab05323 | Rheumatoid arthritis | 6/175 | 61/5900 | 0.0089886 | 0.2031011 | 103226390/103235305/103241278/103236305/103235789/103242710 | IL6/IL11/IL1B/IL15/CXCL1/CCL2 | csab:103226390/csab:103235305/csab:103241278/csab:103236305/csab:103235798/csab:103242710 | 6 | 3 | 103226390/103235305/103236305 | 3 | 103241278/103235789/103242710 |
| csab00600 | Sphingolipid metabolism | 5/175 | 44/5900 | 0.0092319 | 0.2031011 | 103236153/103224742/103233234/103230017/103219011 | UGT8/PLPP3/SMPD3/DEGS1/UGCG | csab:103236153/csab:103224742/csab:103233234/csab:103230017/csab:103219011 | 5 | 3 | 103236153/103230017/103219011 | 2 | 103224742/103233234 |
| csab04625 | C-type lectin receptor signaling pathway | 7/175 | 83/5900 | 0.0111793 | 0.2251985 | 103226390/103241278/103215465/103230198/103216128/103224900/103228406 | IL6/IL1B/EGR3/IKBKE/EGR2/PIK3R3/CBLB | csab:103226390/csab:103241278/csab:103215465/csab:103230198/csab:103216128/csab:103224900/csab:103228406 | 7 | 4 | 103226390/103215465/103216128/103228406 | 3 | 103241278/103230198/103224900 |
| csab05222 | Small cell lung cancer | 7/175 | 87/5900 | 0.0142606 | 0.2251985 | 103224652/103246489/103237430/103214795/103218917/103242634/103224900 | GADD45A/CCND1/MYC/COL4A1/TRAF1/TRAF4/PIK3R3 | csab:103224652/csab:103246489/csab:103237430/csab:103214795/csab:103218917/csab:103242634/csab:103224900 | 7 | 5 | 103224652/103246489/103237430/103214795/103242634 | 2 | 103218917/103224900 |
| csab00900 | Terpenoid backbone biosynthesis | 3/175 | 18/5900 | 0.0150772 | 0.2251985 | 103240824/103215216/103223243 | ACAT2/HMGCS1/HMGCR | csab:103240824/csab:103215216/csab:103223243 | 3 | 3 | 103240824/103215216/103223243 | 0 |  |
| csab04061 | Viral protein interaction with cytokine and cytokine receptor | 5/175 | 50/5900 | 0.0156267 | 0.2251985 | 103226390/103235789/103225587/103223920/103242710 | IL6/CXCL1/TNFRSF1B/IL6R/CCL2 | csab:103226390/csab:103235798/csab:103225587/csab:103223920/csab:103242710 | 5 | 1 | 103226390 | 4 | 103235789/103225587/103223920/103242710 |
| csab00100 | Steroid biosynthesis | 3/175 | 19/5900 | 0.0175208 | 0.2251985 | 103237411/103226567/103215320 | SQLE/CYP51A1/FDFT1 | csab:103237411/csab:103226567/csab:103215320 | 3 | 3 | 103237411/103226567/103215320 | 0 |  |
| csab05169 | Epstein-Barr virus infection | 10/175 | 157/5900 | 0.0178210 | 0.2251985 | 103226390/103224652/103246489/103239154/103230198/103237430/103240296/103224900/103221664/103239156 | IL6/GADD45A/CCND1/OAS1/IKBKE/MYC/TNFAIP3/PIK3R3/LOC103221664/OAS2 | csab:103226390/csab:103224652/csab:103246489/csab:103239154/csab:103230198/csab:103237430/csab:103240296/csab:103224900/csab:103221664/csab:103239156 | 10 | 5 | 103226390/103224652/103246489/103237430/103240296 | 5 | 103239154/103230198/103224900/103221664/103239156 |
| csab04151 | PI3K-Akt signaling pathway | 15/175 | 280/5900 | 0.0185844 | 0.2251985 | 103226390/103238335/103243277/103237005/103235805/103246489/103245773/103235804/103225530/103246479/103237430/103247197/103214795/103224900/103223920 | IL6/NR4A1/ITGB3/IL7/AREG/CCND1/SGK2/EREG/EPHA2/FGFR3/MYC/MCL1/COL4A1/PIK3R3/IL6R | csab:103226390/csab:103238335/csab:103243277/csab:103237005/csab:103235805/csab:103246489/csab:103245773/csab:103235804/csab:103225530/csab:103246479/csab:103237430/csab:103247197/csab:103214795/csab:103224900/csab:103223920 | 15 | 10 | 103226390/103238335/103243277/103235805/103246489/103235804/103225530/103237430/103247197/103214795 | 5 | 103237005/103245773/103246479/103224900/103223920 |
| csab05144 | Malaria | 4/175 | 35/5900 | 0.0191198 | 0.2251985 | 103226390/103241278/103220724/103242710 | IL6/IL1B/SDC1/CCL2 | csab:103226390/csab:103241278/csab:103220724/csab:103242710 | 4 | 1 | 103226390 | 3 | 103241278/103220724/103242710 |
| csab05417 | Lipid and atherosclerosis | 10/175 | 160/5900 | 0.0200779 | 0.2251985 | 103226390/103241278/103240818/103230198/103235789/103234364/103223257/103224900/103223677/103242710 | IL6/IL1B/SOD2/IKBKE/CXCL1/LDLR/NCF4/PIK3R3/HSPA6/CCL2 | csab:103226390/csab:103241278/csab:103240818/csab:103230198/csab:103235798/csab:103234364/csab:103223257/csab:103224900/csab:103223677/csab:103242710 | 10 | 2 | 103226390/103234364 | 8 | 103241278/103240818/103230198/103235789/103223257/103224900/103223677/103242710 |
| csab00770 | Pantothenate and CoA biosynthesis | 3/175 | 20/5900 | 0.0201720 | 0.2251985 | 103218755/103240354/103240351 | BCAT1/LOC103240354/VNN2 | csab:103218755/csab:103240354/csab:103240351 | 3 | 1 | 103218755 | 2 | 103240354/103240351 |
| csab00565 | Ether lipid metabolism | 4/175 | 36/5900 | 0.0210252 | 0.2251985 | 103236153/103235528/103224742/103220573 | UGT8/PLA2G4C/PLPP3/PLB1 | csab:103236153/csab:103235528/csab:103224742/csab:103220573 | 4 | 1 | 103236153 | 3 | 103235528/103224742/103220573 |
| csab05164 | Influenza A | 8/175 | 116/5900 | 0.0213968 | 0.2251985 | 103226390/103241278/103239154/103230198/103224900/103232396/103242710/103239156 | IL6/IL1B/OAS1/IKBKE/PIK3R3/NXF3/CCL2/OAS2 | csab:103226390/csab:103241278/csab:103239154/csab:103230198/csab:103224900/csab:103232396/csab:103242710/csab:103239156 | 8 | 1 | 103226390 | 7 | 103241278/103239154/103230198/103224900/103232396/103242710/103239156 |
| csab04933 | AGE-RAGE signaling pathway in diabetic complications | 7/175 | 95/5900 | 0.0221667 | 0.2251985 | 103226390/103241278/103222156/103246489/103214795/103224900/103242710 | IL6/IL1B/EDN1/CCND1/COL4A1/PIK3R3/CCL2 | csab:103226390/csab:103241278/csab:103222156/csab:103246489/csab:103214795/csab:103224900/csab:103242710 | 7 | 4 | 103226390/103222156/103246489/103214795 | 3 | 103241278/103224900/103242710 |
| csab04610 | Complement and coagulation cascades | 5/175 | 55/5900 | 0.0228053 | 0.2251985 | 103229609/103231401/103215685/103221732/103233571 | SERPINA1/ITGAX/PLAT/LOC103221732/CFD | csab:103229609/csab:103231401/csab:103215685/csab:103221732/csab:103233571 | 5 | 0 |  | 5 | 103229609/103231401/103215685/103221732/103233571 |
| csab00650 | Butanoate metabolism | 3/175 | 21/5900 | 0.0230317 | 0.2251985 | 103240824/103215216/103227954 | ACAT2/HMGCS1/ABAT | csab:103240824/csab:103215216/csab:103227954 | 3 | 2 | 103240824/103215216 | 1 | 103227954 |
| csab05219 | Bladder cancer | 4/175 | 38/5900 | 0.0251752 | 0.2318612 | 103244654/103246489/103246479/103237430 | HBEGF/CCND1/FGFR3/MYC | csab:103244654/csab:103246489/csab:103246479/csab:103237430 | 4 | 3 | 103244654/103246489/103237430 | 1 | 103246479 |
| csab01230 | Biosynthesis of amino acids | 5/175 | 57/5900 | 0.0261887 | 0.2318612 | 103242625/103218755/103224624/103218486/103224094 | ALDOC/BCAT1/CTH/ENO2/PHGDH | csab:103242625/csab:103218755/csab:103224624/csab:103218486/csab:103224094 | 5 | 3 | 103218755/103224624/103224094 | 2 | 103242625/103218486 |
| csab05200 | Pathways in cancer | 20/175 | 428/5900 | 0.0275820 | 0.2318612 | 103226390/103220342/103246725/103237005/103224652/103230197/103222156/103246489/103223483/103236305/103246479/103237430/103224207/103214795/103218917/103218702/103242634/103224900/103239621/103223920 | IL6/EPAS1/BMP2/IL7/GADD45A/RASSF5/EDN1/CCND1/WNT7B/IL15/FGFR3/MYC/WNT2B/COL4A1/TRAF1/MGST1/TRAF4/PIK3R3/NOTCH1/IL6R | csab:103226390/csab:103220342/csab:103246725/csab:103237005/csab:103224652/csab:103230197/csab:103222156/csab:103246489/csab:103223483/csab:103236305/csab:103246479/csab:103237430/csab:103224207/csab:103214795/csab:103218917/csab:103218702/csab:103242634/csab:103224900/csab:103239621/csab:103223920 | 20 | 10 | 103226390/103246725/103224652/103222156/103246489/103236305/103237430/103214795/103218702/103242634 | 10 | 103220342/103237005/103230197/103223483/103246479/103224207/103218917/103224900/103239621/103223920 |
| csab05206 | MicroRNAs in cancer | 9/175 | 145/5900 | 0.0278436 | 0.2318612 | 103214617/103243277/103246489/103246479/103237430/103247197/103224900/103239621/103214788 | SPRY2/ITGB3/CCND1/FGFR3/MYC/MCL1/PIK3R3/NOTCH1/IRS2 | csab:103214617/csab:103243277/csab:103246489/csab:103246479/csab:103237430/csab:103247197/csab:103224900/csab:103239621/csab:103214788 | 9 | 6 | 103214617/103243277/103246489/103237430/103247197/103214788 | 3 | 103246479/103224900/103239621 |
| csab05205 | Proteoglycans in cancer | 10/175 | 169/5900 | 0.0281044 | 0.2318612 | 103243277/103244654/103246489/103223483/103218491/103237430/103220724/103224207/103218213/103224900 | ITGB3/HBEGF/CCND1/WNT7B/PTPN6/MYC/SDC1/WNT2B/GPC1/PIK3R3 | csab:103243277/csab:103244654/csab:103246489/csab:103223483/csab:103218491/csab:103237430/csab:103220724/csab:103224207/csab:103218213/csab:103224900 | 10 | 4 | 103243277/103244654/103246489/103237430 | 6 | 103223483/103218491/103220724/103224207/103218213/103224900 |
| csab04012 | ErbB signaling pathway | 6/175 | 79/5900 | 0.0291478 | 0.2331824 | 103235805/103244654/103235804/103237430/103224900/103228406 | AREG/HBEGF/EREG/MYC/PIK3R3/CBLB | csab:103235805/csab:103244654/csab:103235804/csab:103237430/csab:103224900/csab:103228406 | 6 | 5 | 103235805/103244654/103235804/103237430/103228406 | 1 | 103224900 |
| csab04010 | MAPK signaling pathway | 13/175 | 247/5900 | 0.0312409 | 0.2378846 | 103238335/103241278/103216504/103235805/103224652/103235804/103225530/103246479/103237430/103217090/103235528/103225290/103223677 | NR4A1/IL1B/DUSP5/AREG/GADD45A/EREG/EPHA2/FGFR3/MYC/CACNB4/PLA2G4C/MAP3K6/HSPA6 | csab:103238335/csab:103241278/csab:103216504/csab:103235805/csab:103224652/csab:103235804/csab:103225530/csab:103246479/csab:103237430/csab:103217090/csab:103235528/csab:103225290/csab:103223677 | 13 | 8 | 103238335/103216504/103235805/103224652/103235804/103225530/103237430/103217090 | 5 | 103241278/103246479/103235528/103225290/103223677 |
| csab05160 | Hepatitis C | 8/175 | 125/5900 | 0.0317119 | 0.2378846 | 103246489/103239154/103230198/103237430/103234364/103241901/103224900/103239156 | CCND1/OAS1/IKBKE/MYC/LDLR/CLDN1/PIK3R3/OAS2 | csab:103246489/csab:103239154/csab:103230198/csab:103237430/csab:103234364/csab:103241901/csab:103224900/csab:103239156 | 8 | 4 | 103246489/103237430/103234364/103241901 | 4 | 103239154/103230198/103224900/103239156 |
| csab05210 | Colorectal cancer | 6/175 | 81/5900 | 0.0324388 | 0.2378846 | 103235805/103224652/103246489/103235804/103237430/103224900 | AREG/GADD45A/CCND1/EREG/MYC/PIK3R3 | csab:103235805/csab:103224652/csab:103246489/csab:103235804/csab:103237430/csab:103224900 | 6 | 5 | 103235805/103224652/103246489/103235804/103237430 | 1 | 103224900 |
| csab04919 | Thyroid hormone signaling pathway | 7/175 | 105/5900 | 0.0357750 | 0.2498488 | 103243277/103232196/103246489/103237335/103237430/103224900/103239621 | ITGB3/SLC16A2/CCND1/MED30/MYC/PIK3R3/NOTCH1 | csab:103243277/csab:103232196/csab:103246489/csab:103237335/csab:103237430/csab:103224900/csab:103239621 | 7 | 4 | 103243277/103246489/103237335/103237430 | 3 | 103232196/103224900/103239621 |
| csab04066 | HIF-1 signaling pathway | 6/175 | 83/5900 | 0.0359631 | 0.2498488 | 103226390/103242625/103222156/103218486/103224900/103223920 | IL6/ALDOC/EDN1/ENO2/PIK3R3/IL6R | csab:103226390/csab:103242625/csab:103222156/csab:103218486/csab:103224900/csab:103223920 | 6 | 2 | 103226390/103222156 | 4 | 103242625/103218486/103224900/103223920 |
| csab00970 | Aminoacyl-tRNA biosynthesis | 4/175 | 43/5900 | 0.0375812 | 0.2543957 | 103226274/103219751/103229694/103238568 | GARS1/IARS1/WARS1/MARS1 | csab:103226274/csab:103219751/csab:103229694/csab:103238568 | 4 | 4 | 103226274/103219751/103229694/103238568 | 0 |  |
| csab05134 | Legionellosis | 4/175 | 44/5900 | 0.0404161 | 0.2667464 | 103226390/103241278/103235789/103223677 | IL6/IL1B/CXCL1/HSPA6 | csab:103226390/csab:103241278/csab:103235798/csab:103223677 | 4 | 1 | 103226390 | 3 | 103241278/103235789/103223677 |
| csab00280 | Valine, leucine and isoleucine degradation | 4/175 | 46/5900 | 0.0464417 | 0.2990393 | 103218755/103240824/103215216/103227954 | BCAT1/ACAT2/HMGCS1/ABAT | csab:103218755/csab:103240824/csab:103215216/csab:103227954 | 4 | 3 | 103218755/103240824/103215216 | 1 | 103227954 |
| csab04310 | Wnt signaling pathway | 8/175 | 141/5900 | 0.0575898 | 0.3577567 | 103232583/103246489/103223483/103237430/103215672/103231576/103224207/103237471 | GPC4/CCND1/WNT7B/MYC/SFRP1/TBL1X/WNT2B/CCN4 | csab:103232583/csab:103246489/csab:103223483/csab:103237430/csab:103215672/csab:103231576/csab:103224207/csab:103237471 | 8 | 4 | 103232583/103246489/103237430/103231576 | 4 | 103223483/103215672/103224207/103237471 |
| csab04068 | FoxO signaling pathway | 7/175 | 117/5900 | 0.0582710 | 0.3577567 | 103226390/103240818/103224652/103246489/103245773/103224900/103214788 | IL6/SOD2/GADD45A/CCND1/SGK2/PIK3R3/IRS2 | csab:103226390/csab:103240818/csab:103224652/csab:103246489/csab:103245773/csab:103224900/csab:103214788 | 7 | 4 | 103226390/103224652/103246489/103214788 | 3 | 103240818/103245773/103224900 |
| csab04672 | Intestinal immune network for IgA production | 3/175 | 31/5900 | 0.0628502 | 0.3692413 | 103226390/103230184/103236305 | IL6/PIGR/IL15 | csab:103226390/csab:103230184/csab:103236305 | 3 | 2 | 103226390/103236305 | 1 | 103230184 |
| csab05410 | Hypertrophic cardiomyopathy | 5/175 | 74/5900 | 0.0677883 | 0.3692413 | 103226390/103243277/103222156/103217090/103219220 | IL6/ITGB3/EDN1/CACNB4/TPM2 | csab:103226390/csab:103243277/csab:103222156/csab:103217090/csab:103219220 | 5 | 4 | 103226390/103243277/103222156/103217090 | 1 | 103219220 |
| csab05216 | Thyroid cancer | 3/175 | 32/5900 | 0.0678973 | 0.3692413 | 103224652/103246489/103237430 | GADD45A/CCND1/MYC | csab:103224652/csab:103246489/csab:103237430 | 3 | 3 | 103224652/103246489/103237430 | 0 |  |
| csab05146 | Amoebiasis | 5/175 | 75/5900 | 0.0709700 | 0.3692413 | 103226390/103241278/103235789/103214795/103224900 | IL6/IL1B/CXCL1/COL4A1/PIK3R3 | csab:103226390/csab:103241278/csab:103235798/csab:103214795/csab:103224900 | 5 | 2 | 103226390/103214795 | 3 | 103241278/103235789/103224900 |
| csab05163 | Human cytomegalovirus infection | 9/175 | 173/5900 | 0.0709881 | 0.3692413 | 103226390/103241278/103243277/103246489/103237430/103224900/103221664/103223920/103242710 | IL6/IL1B/ITGB3/CCND1/MYC/PIK3R3/LOC103221664/IL6R/CCL2 | csab:103226390/csab:103241278/csab:103243277/csab:103246489/csab:103237430/csab:103224900/csab:103221664/csab:103223920/csab:103242710 | 9 | 4 | 103226390/103243277/103246489/103237430 | 5 | 103241278/103224900/103221664/103223920/103242710 |
| csab04213 | Longevity regulating pathway - multiple species | 4/175 | 53/5900 | 0.0712284 | 0.3692413 | 103240818/103224900/103214788/103223677 | SOD2/PIK3R3/IRS2/HSPA6 | csab:103240818/csab:103224900/csab:103214788/csab:103223677 | 4 | 1 | 103214788 | 3 | 103240818/103224900/103223677 |
| csab00450 | Selenocompound metabolism | 2/175 | 15/5900 | 0.0713307 | 0.3692413 | 103224624/103238568 | CTH/MARS1 | csab:103224624/csab:103238568 | 2 | 2 | 103224624/103238568 | 0 |  |
| csab00592 | alpha-Linolenic acid metabolism | 2/175 | 15/5900 | 0.0713307 | 0.3692413 | 103235528/103220573 | PLA2G4C/PLB1 | csab:103235528/csab:103220573 | 2 | 0 |  | 2 | 103235528/103220573 |
| csab05224 | Breast cancer | 7/175 | 125/5900 | 0.0772558 | 0.3770349 | 103224652/103246489/103223483/103237430/103224207/103224900/103239621 | GADD45A/CCND1/WNT7B/MYC/WNT2B/PIK3R3/NOTCH1 | csab:103224652/csab:103246489/csab:103223483/csab:103237430/csab:103224207/csab:103224900/csab:103239621 | 7 | 3 | 103224652/103246489/103237430 | 4 | 103223483/103224207/103224900/103239621 |
| csab04666 | Fc gamma R-mediated phagocytosis | 5/175 | 77/5900 | 0.0775765 | 0.3770349 | 103240591/103235528/103224742/103224900/103224044 | WASF1/PLA2G4C/PLPP3/PIK3R3/PIP5K1A | csab:103240591/csab:103235528/csab:103224742/csab:103224900/csab:103224044 | 5 | 2 | 103240591/103224044 | 3 | 103235528/103224742/103224900 |
| csab04936 | Alcoholic liver disease | 6/175 | 101/5900 | 0.0787088 | 0.3770349 | 103226390/103241278/103221732/103246489/103230198/103235789 | IL6/IL1B/LOC103221732/CCND1/IKBKE/CXCL1 | csab:103226390/csab:103241278/csab:103221732/csab:103246489/csab:103230198/csab:103235798 | 6 | 2 | 103226390/103246489 | 4 | 103241278/103221732/103230198/103235789 |
| csab05213 | Endometrial cancer | 4/175 | 55/5900 | 0.0793403 | 0.3770349 | 103224652/103246489/103237430/103224900 | GADD45A/CCND1/MYC/PIK3R3 | csab:103224652/csab:103246489/csab:103237430/csab:103224900 | 4 | 3 | 103224652/103246489/103237430 | 1 | 103224900 |
| csab00591 | Linoleic acid metabolism | 2/175 | 16/5900 | 0.0799771 | 0.3770349 | 103235528/103220573 | PLA2G4C/PLB1 | csab:103235528/csab:103220573 | 2 | 0 |  | 2 | 103235528/103220573 |
| csab05217 | Basal cell carcinoma | 4/175 | 56/5900 | 0.0835619 | 0.3781192 | 103246725/103224652/103223483/103224207 | BMP2/GADD45A/WNT7B/WNT2B | csab:103246725/csab:103224652/csab:103223483/csab:103224207 | 4 | 2 | 103246725/103224652 | 2 | 103223483/103224207 |
| csab00830 | Retinol metabolism | 3/175 | 35/5900 | 0.0840918 | 0.3781192 | 103236970/103225584/103231285 | RDH10/DHRS3/ALDH1A3 | csab:103236970/csab:103225584/csab:103231285 | 3 | 1 | 103231285 | 2 | 103236970/103225584 |
| csab04064 | NF-kappa B signaling pathway | 5/175 | 79/5900 | 0.0845039 | 0.3781192 | 103241278/103224652/103235789/103218917/103240296 | IL1B/GADD45A/CXCL1/TRAF1/TNFAIP3 | csab:103241278/csab:103224652/csab:103235798/csab:103218917/csab:103240296 | 5 | 2 | 103224652/103240296 | 3 | 103241278/103235789/103218917 |
| csab04913 | Ovarian steroidogenesis | 3/175 | 36/5900 | 0.0898251 | 0.3952304 | 103234364/103235528/103237812 | LDLR/PLA2G4C/LOC103237812 | csab:103234364/csab:103235528/csab:103237812 | 3 | 2 | 103234364/103237812 | 1 | 103235528 |
| csab04210 | Apoptosis | 6/175 | 107/5900 | 0.0974392 | 0.4179726 | 103224652/103247197/103218917/103245241/103224900/103235922 | GADD45A/MCL1/TRAF1/CTSH/PIK3R3/PTPN13 | csab:103224652/csab:103247197/csab:103218917/csab:103245241/csab:103224900/csab:103235922 | 6 | 3 | 103224652/103247197/103235922 | 3 | 103218917/103245241/103224900 |
| csab05332 | Graft-versus-host disease | 2/175 | 18/5900 | 0.0981602 | 0.4179726 | 103226390/103241278 | IL6/IL1B | csab:103226390/csab:103241278 | 2 | 1 | 103226390 | 1 | 103241278 |
| csab05230 | Central carbon metabolism in cancer | 4/175 | 60/5900 | 0.1015159 | 0.4227138 | 103246479/103237430/103224900/103233435 | FGFR3/MYC/PIK3R3/SLC7A5 | csab:103246479/csab:103237430/csab:103224900/csab:103233435 | 4 | 2 | 103237430/103233435 | 2 | 103246479/103224900 |
| csab04218 | Cellular senescence | 7/175 | 134/5900 | 0.1024761 | 0.4227138 | 103226390/103224652/103230197/103246489/103237430/103237062/103224900 | IL6/GADD45A/RASSF5/CCND1/MYC/E2F5/PIK3R3 | csab:103226390/csab:103224652/csab:103230197/csab:103246489/csab:103237430/csab:103237062/csab:103224900 | 7 | 5 | 103226390/103224652/103246489/103237430/103237062 | 2 | 103230197/103224900 |
| csab04115 | p53 signaling pathway | 4/175 | 61/5900 | 0.1062623 | 0.4253916 | 103225266/103224652/103246489/103236967 | SESN2/GADD45A/CCND1/CD82 | csab:103225266/csab:103224652/csab:103246489/csab:103236967 | 4 | 3 | 103225266/103224652/103246489 | 1 | 103236967 |
| csab05231 | Choline metabolism in cancer | 5/175 | 85/5900 | 0.1071603 | 0.4253916 | 103240591/103235528/103224742/103224900/103224044 | WASF1/PLA2G4C/PLPP3/PIK3R3/PIP5K1A | csab:103240591/csab:103235528/csab:103224742/csab:103224900/csab:103224044 | 5 | 2 | 103240591/103224044 | 3 | 103235528/103224742/103224900 |
| csab04623 | Cytosolic DNA-sensing pathway | 3/175 | 39/5900 | 0.1079592 | 0.4253916 | 103226390/103241278/103230198 | IL6/IL1B/IKBKE | csab:103226390/csab:103241278/csab:103230198 | 3 | 1 | 103226390 | 2 | 103241278/103230198 |
| csab05223 | Non-small cell lung cancer | 4/175 | 63/5900 | 0.1160510 | 0.4505511 | 103224652/103230197/103246489/103224900 | GADD45A/RASSF5/CCND1/PIK3R3 | csab:103224652/csab:103230197/csab:103246489/csab:103224900 | 4 | 2 | 103224652/103246489 | 2 | 103230197/103224900 |
| csab04930 | Type II diabetes mellitus | 3/175 | 41/5900 | 0.1207738 | 0.4620909 | 103238870/103224900/103214788 | SOCS2/PIK3R3/IRS2 | csab:103238870/csab:103224900/csab:103214788 | 3 | 2 | 103238870/103214788 | 1 | 103224900 |
| csab04940 | Type I diabetes mellitus | 2/175 | 21/5900 | 0.1272967 | 0.4622311 | 103241278/103217907 | IL1B/PTPRN | csab:103241278/csab:103217907 | 2 | 0 |  | 2 | 103241278/103217907 |
| csab05143 | African trypanosomiasis | 2/175 | 21/5900 | 0.1272967 | 0.4622311 | 103226390/103241278 | IL6/IL1B | csab:103226390/csab:103241278 | 2 | 1 | 103226390 | 1 | 103241278 |
| csab00270 | Cysteine and methionine metabolism | 3/175 | 42/5900 | 0.1273821 | 0.4622311 | 103218755/103224624/103224094 | BCAT1/CTH/PHGDH | csab:103218755/csab:103224624/csab:103224094 | 3 | 3 | 103218755/103224624/103224094 | 0 |  |
| csab04390 | Hippo signaling pathway | 7/175 | 142/5900 | 0.1282329 | 0.4622311 | 103246725/103248148/103235805/103246489/103223483/103237430/103224207 | BMP2/DLG2/AREG/CCND1/WNT7B/MYC/WNT2B | csab:103246725/csab:103248148/csab:103235805/csab:103246489/csab:103223483/csab:103237430/csab:103224207 | 7 | 5 | 103246725/103248148/103235805/103246489/103237430 | 2 | 103223483/103224207 |
| csab04550 | Signaling pathways regulating pluripotency of stem cells | 6/175 | 116/5900 | 0.1295648 | 0.4622311 | 103219053/103223483/103246479/103237430/103224207/103224900 | KLF4/WNT7B/FGFR3/MYC/WNT2B/PIK3R3 | csab:103219053/csab:103223483/csab:103246479/csab:103237430/csab:103224207/csab:103224900 | 6 | 2 | 103219053/103237430 | 4 | 103223483/103246479/103224207/103224900 |
| csab05207 | Chemical carcinogenesis - receptor activation | 7/175 | 146/5900 | 0.1422333 | 0.4933536 | 103219053/103246489/103214580/103230198/103237430/103218702/103224900 | KLF4/CCND1/KLF5/IKBKE/MYC/MGST1/PIK3R3 | csab:103219053/csab:103246489/csab:103214580/csab:103230198/csab:103237430/csab:103218702/csab:103224900 | 7 | 5 | 103219053/103246489/103214580/103237430/103218702 | 2 | 103230198/103224900 |
| csab05225 | Hepatocellular carcinoma | 7/175 | 146/5900 | 0.1422333 | 0.4933536 | 103224652/103246489/103223483/103237430/103224207/103218702/103224900 | GADD45A/CCND1/WNT7B/MYC/WNT2B/MGST1/PIK3R3 | csab:103224652/csab:103246489/csab:103223483/csab:103237430/csab:103224207/csab:103218702/csab:103224900 | 7 | 4 | 103224652/103246489/103237430/103218702 | 3 | 103223483/103224207/103224900 |
| csab01521 | EGFR tyrosine kinase inhibitor resistance | 4/175 | 69/5900 | 0.1476269 | 0.4933536 | 103226390/103246479/103224900/103223920 | IL6/FGFR3/PIK3R3/IL6R | csab:103226390/csab:103246479/csab:103224900/csab:103223920 | 4 | 1 | 103226390 | 3 | 103246479/103224900/103223920 |
| csab04975 | Fat digestion and absorption | 2/175 | 23/5900 | 0.1477059 | 0.4933536 | 103224742/103240824 | PLPP3/ACAT2 | csab:103224742/csab:103240824 | 2 | 1 | 103240824 | 1 | 103224742 |
| csab00980 | Metabolism of xenobiotics by cytochrome P450 | 3/175 | 45/5900 | 0.1479451 | 0.4933536 | 103230154/103218702/103218549 | HSD11B1/MGST1/CBR3 | csab:103230154/csab:103218702/csab:103218549 | 3 | 1 | 103218702 | 2 | 103230154/103218549 |
| csab05202 | Transcriptional misregulation in cancer | 7/175 | 148/5900 | 0.1495011 | 0.4933536 | 103226390/103215685/103219133/103224652/103228427/103237430/103218917 | IL6/PLAT/NR4A3/GADD45A/NFKBIZ/MYC/TRAF1 | csab:103226390/csab:103215685/csab:103219133/csab:103224652/csab:103228427/csab:103237430/csab:103218917 | 7 | 5 | 103226390/103219133/103224652/103228427/103237430 | 2 | 103215685/103218917 |
| csab00601 | Glycosphingolipid biosynthesis - lacto and neolacto series | 2/175 | 24/5900 | 0.1581465 | 0.5113437 | 103234996/103217238 | FUT1/B3GALT1 | csab:103234996/csab:103217238 | 2 | 2 | 103234996/103217238 | 0 |  |
| csab05220 | Chronic myeloid leukemia | 4/175 | 71/5900 | 0.1588264 | 0.5113437 | 103224652/103246489/103237430/103224900 | GADD45A/CCND1/MYC/PIK3R3 | csab:103224652/csab:103246489/csab:103237430/csab:103224900 | 4 | 3 | 103224652/103246489/103237430 | 1 | 103224900 |
| csab01523 | Antifolate resistance | 2/175 | 25/5900 | 0.1687203 | 0.5279192 | 103226390/103241278 | IL6/IL1B | csab:103226390/csab:103241278 | 2 | 1 | 103226390 | 1 | 103241278 |
| csab04270 | Vascular smooth muscle contraction | 5/175 | 99/5900 | 0.1699740 | 0.5279192 | 103222156/103239748/103235528/103240389/103223064 | EDN1/CALCB/PLA2G4C/ADM/ADORA2A | csab:103222156/csab:103239748/csab:103235528/csab:103240389/csab:103223064 | 5 | 2 | 103222156/103239748 | 3 | 103235528/103240389/103223064 |
| csab04931 | Insulin resistance | 5/175 | 99/5900 | 0.1699740 | 0.5279192 | 103226390/103247816/103224900/103214788/103220112 | IL6/TRIB3/PIK3R3/IRS2/GFPT1 | csab:103226390/csab:103247816/csab:103224900/csab:103214788/csab:103220112 | 5 | 4 | 103226390/103247816/103214788/103220112 | 1 | 103224900 |
| csab05226 | Gastric cancer | 6/175 | 127/5900 | 0.1748153 | 0.5366422 | 103224652/103246489/103223483/103237430/103224207/103224900 | GADD45A/CCND1/WNT7B/MYC/WNT2B/PIK3R3 | csab:103224652/csab:103246489/csab:103223483/csab:103237430/csab:103224207/csab:103224900 | 6 | 3 | 103224652/103246489/103237430 | 3 | 103223483/103224207/103224900 |
| csab04620 | Toll-like receptor signaling pathway | 4/175 | 75/5900 | 0.1821176 | 0.5526326 | 103226390/103241278/103230198/103224900 | IL6/IL1B/IKBKE/PIK3R3 | csab:103226390/csab:103241278/csab:103230198/csab:103224900 | 4 | 1 | 103226390 | 3 | 103241278/103230198/103224900 |
| csab04211 | Longevity regulating pathway | 4/175 | 76/5900 | 0.1881111 | 0.5641902 | 103240818/103225266/103224900/103214788 | SOD2/SESN2/PIK3R3/IRS2 | csab:103240818/csab:103225266/csab:103224900/csab:103214788 | 4 | 2 | 103225266/103214788 | 2 | 103240818/103224900 |
| csab04960 | Aldosterone-regulated sodium reabsorption | 2/175 | 27/5900 | 0.1902005 | 0.5641902 | 103224900/103229709 | PIK3R3/SCNN1B | csab:103224900/csab:103229709 | 2 | 0 |  | 2 | 103224900/103229709 |
| csab04350 | TGF-beta signaling pathway | 4/175 | 77/5900 | 0.1941678 | 0.5695590 | 103246725/103237430/103237062/103245923 | BMP2/MYC/E2F5/GREM1 | csab:103246725/csab:103237430/csab:103237062/csab:103245923 | 4 | 4 | 103246725/103237430/103237062/103245923 | 0 |  |
| csab04150 | mTOR signaling pathway | 6/175 | 132/5900 | 0.1972879 | 0.5723517 | 103225266/103223483/103224207/103246611/103224900/103233435 | SESN2/WNT7B/WNT2B/CASTOR2/PIK3R3/SLC7A5 | csab:103225266/csab:103223483/csab:103224207/csab:103246611/csab:103224900/csab:103233435 | 6 | 2 | 103225266/103233435 | 4 | 103223483/103224207/103246611/103224900 |
| csab00250 | Alanine, aspartate and glutamate metabolism | 2/175 | 28/5900 | 0.2010756 | 0.5769996 | 103227954/103220112 | ABAT/GFPT1 | csab:103227954/csab:103220112 | 2 | 1 | 103220112 | 1 | 103227954 |
| csab05133 | Pertussis | 3/175 | 53/5900 | 0.2072127 | 0.5882166 | 103226390/103241278/103221732 | IL6/IL1B/LOC103221732 | csab:103226390/csab:103241278/csab:103221732 | 3 | 1 | 103226390 | 2 | 103241278/103221732 |
| csab00564 | Glycerophospholipid metabolism | 4/175 | 80/5900 | 0.2126902 | 0.5973426 | 103235528/103228224/103224742/103220573 | PLA2G4C/PLA1A/PLPP3/PLB1 | csab:103235528/csab:103228224/csab:103224742/csab:103220573 | 4 | 0 |  | 4 | 103235528/103228224/103224742/103220573 |
| csab05140 | Leishmaniasis | 3/175 | 54/5900 | 0.2149764 | 0.5974082 | 103241278/103218491/103223257 | IL1B/PTPN6/NCF4 | csab:103241278/csab:103218491/csab:103223257 | 3 | 0 |  | 3 | 103241278/103218491/103223257 |
| csab05161 | Hepatitis B | 6/175 | 138/5900 | 0.2255904 | 0.6203736 | 103226390/103215465/103230198/103237430/103216128/103224900 | IL6/EGR3/IKBKE/MYC/EGR2/PIK3R3 | csab:103226390/csab:103215465/csab:103230198/csab:103237430/csab:103216128/csab:103224900 | 6 | 4 | 103226390/103215465/103237430/103216128 | 2 | 103230198/103224900 |
| csab05135 | Yersinia infection | 5/175 | 112/5900 | 0.2382705 | 0.6428021 | 103226390/103241278/103224900/103224044/103242710 | IL6/IL1B/PIK3R3/PIP5K1A/CCL2 | csab:103226390/csab:103241278/csab:103224900/csab:103224044/csab:103242710 | 5 | 2 | 103226390/103224044 | 3 | 103241278/103224900/103242710 |
| csab05221 | Acute myeloid leukemia | 3/175 | 57/5900 | 0.2386159 | 0.6428021 | 103246489/103237430/103224900 | CCND1/MYC/PIK3R3 | csab:103246489/csab:103237430/csab:103224900 | 3 | 2 | 103246489/103237430 | 1 | 103224900 |
| csab05218 | Melanoma | 3/175 | 58/5900 | 0.2465952 | 0.6575873 | 103224652/103246489/103224900 | GADD45A/CCND1/PIK3R3 | csab:103224652/csab:103246489/csab:103224900 | 3 | 2 | 103224652/103246489 | 1 | 103224900 |
| csab05142 | Chagas disease | 4/175 | 86/5900 | 0.2510829 | 0.6628588 | 103226390/103241278/103224900/103242710 | IL6/IL1B/PIK3R3/CCL2 | csab:103226390/csab:103241278/csab:103224900/csab:103242710 | 4 | 1 | 103226390 | 3 | 103241278/103224900/103242710 |
| csab01522 | Endocrine resistance | 4/175 | 87/5900 | 0.2576259 | 0.6732618 | 103244654/103246489/103224900/103239621 | HBEGF/CCND1/PIK3R3/NOTCH1 | csab:103244654/csab:103246489/csab:103224900/csab:103239621 | 4 | 2 | 103244654/103246489 | 2 | 103224900/103239621 |
| csab04721 | Synaptic vesicle cycle | 3/175 | 60/5900 | 0.2626741 | 0.6732618 | 103246629/103246502/103239806 | STX1A/SNAP25/DNM1 | csab:103246629/csab:103246502/csab:103239806 | 3 | 0 |  | 3 | 103246629/103246502/103239806 |
| csab04917 | Prolactin signaling pathway | 3/175 | 60/5900 | 0.2626741 | 0.6732618 | 103246489/103238870/103224900 | CCND1/SOCS2/PIK3R3 | csab:103246489/csab:103238870/csab:103224900 | 3 | 2 | 103246489/103238870 | 1 | 103224900 |
| csab01200 | Carbon metabolism | 4/175 | 89/5900 | 0.2708124 | 0.6874467 | 103242625/103218486/103224094/103240824 | ALDOC/ENO2/PHGDH/ACAT2 | csab:103242625/csab:103218486/csab:103224094/csab:103240824 | 4 | 2 | 103224094/103240824 | 2 | 103242625/103218486 |
| csab05167 | Kaposi sarcoma-associated herpesvirus infection | 6/175 | 148/5900 | 0.2753968 | 0.6924261 | 103226390/103246489/103230198/103235789/103237430/103224900 | IL6/CCND1/IKBKE/CXCL1/MYC/PIK3R3 | csab:103226390/csab:103246489/csab:103230198/csab:103235798/csab:103237430/csab:103224900 | 6 | 3 | 103226390/103246489/103237430 | 3 | 103230198/103235789/103224900 |
| csab05214 | Glioma | 3/175 | 62/5900 | 0.2788825 | 0.6945752 | 103224652/103246489/103224900 | GADD45A/CCND1/PIK3R3 | csab:103224652/csab:103246489/csab:103224900 | 3 | 2 | 103224652/103246489 | 1 | 103224900 |
| csab00430 | Taurine and hypotaurine metabolism | 1/175 | 11/5900 | 0.2821489 | 0.6961431 | 103230592 | FMO4 | csab:103230592 | 1 | 0 |  | 1 | 103230592 |
| csab00982 | Drug metabolism - cytochrome P450 | 2/175 | 36/5900 | 0.2895577 | 0.6967741 | 103218702/103230592 | MGST1/FMO4 | csab:103218702/csab:103230592 | 2 | 1 | 103218702 | 1 | 103230592 |
| csab04979 | Cholesterol metabolism | 2/175 | 36/5900 | 0.2895577 | 0.6967741 | 103234364/103229330 | LDLR/NPC2 | csab:103234364/csab:103229330 | 2 | 2 | 103234364/103229330 | 0 |  |
| csab04810 | Regulation of actin cytoskeleton | 7/175 | 181/5900 | 0.2903225 | 0.6967741 | 103231401/103243277/103240591/103246479/103224900/103223856/103224044 | ITGAX/ITGB3/WASF1/FGFR3/PIK3R3/IQGAP3/PIP5K1A | csab:103231401/csab:103243277/csab:103240591/csab:103246479/csab:103224900/csab:103223856/csab:103224044 | 7 | 3 | 103243277/103240591/103224044 | 4 | 103231401/103246479/103224900/103223856 |
| csab04911 | Insulin secretion | 3/175 | 64/5900 | 0.2951848 | 0.7020611 | 103226636/103246629/103246502 | PCLO/STX1A/SNAP25 | csab:103226636/csab:103246629/csab:103246502 | 3 | 0 |  | 3 | 103226636/103246629/103246502 |
| csab00260 | Glycine, serine and threonine metabolism | 2/175 | 37/5900 | 0.3006342 | 0.7032838 | 103224624/103224094 | CTH/PHGDH | csab:103224624/csab:103224094 | 2 | 2 | 103224624/103224094 | 0 |  |
| csab04014 | Ras signaling pathway | 7/175 | 184/5900 | 0.3045586 | 0.7032838 | 103230197/103225530/103246479/103235528/103228224/103224900/103223351 | RASSF5/EPHA2/FGFR3/PLA2G4C/PLA1A/PIK3R3/RIN1 | csab:103230197/csab:103225530/csab:103246479/csab:103235528/csab:103228224/csab:103224900/csab:103223351 | 7 | 1 | 103225530 | 6 | 103230197/103246479/103235528/103228224/103224900/103223351 |
| csab05100 | Bacterial invasion of epithelial cells | 3/175 | 66/5900 | 0.3115471 | 0.7032838 | 103240591/103224900/103239806 | WASF1/PIK3R3/DNM1 | csab:103240591/csab:103224900/csab:103239806 | 3 | 1 | 103240591 | 2 | 103224900/103239806 |
| csab00380 | Tryptophan metabolism | 2/175 | 38/5900 | 0.3116826 | 0.7032838 | 103240824/103232876 | ACAT2/LOC103232876 | csab:103240824/csab:103232876 | 2 | 1 | 103240824 | 1 | 103232876 |
| csab04961 | Endocrine and other factor-regulated calcium reabsorption | 2/175 | 38/5900 | 0.3116826 | 0.7032838 | 103238835/103239806 | ATP2B1/DNM1 | csab:103238835/csab:103239806 | 2 | 1 | 103238835 | 1 | 103239806 |
| csab05204 | Chemical carcinogenesis - DNA adducts | 2/175 | 38/5900 | 0.3116826 | 0.7032838 | 103230154/103218702 | HSD11B1/MGST1 | csab:103230154/csab:103218702 | 2 | 1 | 103218702 | 1 | 103230154 |
| csab04727 | GABAergic synapse | 3/175 | 67/5900 | 0.3197406 | 0.7138212 | 103238195/103227954/103238196 | SLC38A1/ABAT/SLC38A2 | csab:103238195/csab:103227954/csab:103238196 | 3 | 2 | 103238195/103238196 | 1 | 103227954 |
| csab00140 | Steroid hormone biosynthesis | 2/175 | 39/5900 | 0.3226947 | 0.7138212 | 103230154/103237812 | HSD11B1/LOC103237812 | csab:103230154/csab:103237812 | 2 | 1 | 103237812 | 1 | 103230154 |
| csab04934 | Cushing syndrome | 5/175 | 127/5900 | 0.3244642 | 0.7138212 | 103238335/103246489/103223483/103234364/103224207 | NR4A1/CCND1/WNT7B/LDLR/WNT2B | csab:103238335/csab:103246489/csab:103223483/csab:103234364/csab:103224207 | 5 | 3 | 103238335/103246489/103234364 | 2 | 103223483/103224207 |
| csab04072 | Phospholipase D signaling pathway | 5/175 | 128/5900 | 0.3303683 | 0.7208036 | 103235528/103224742/103224900/103224044/103239806 | PLA2G4C/PLPP3/PIK3R3/PIP5K1A/DNM1 | csab:103235528/csab:103224742/csab:103224900/csab:103224044/csab:103239806 | 5 | 1 | 103224044 | 4 | 103235528/103224742/103224900/103239806 |
| csab03018 | RNA degradation | 3/175 | 69/5900 | 0.3361332 | 0.7272139 | 103218486/103242916/103234733 | ENO2/TOB1/EXOSC5 | csab:103218486/csab:103242916/csab:103234733 | 3 | 2 | 103242916/103234733 | 1 | 103218486 |
| csab01210 | 2-Oxocarboxylic acid metabolism | 1/175 | 14/5900 | 0.3442721 | 0.7272139 | 103218755 | BCAT1 | csab:103218755 | 1 | 1 | 103218755 | 0 |  |
| csab04977 | Vitamin digestion and absorption | 1/175 | 14/5900 | 0.3442721 | 0.7272139 | 103220573 | PLB1 | csab:103220573 | 1 | 0 |  | 1 | 103220573 |
| csab04925 | Aldosterone synthesis and secretion | 3/175 | 70/5900 | 0.3443248 | 0.7272139 | 103238335/103234364/103238835 | NR4A1/LDLR/ATP2B1 | csab:103238335/csab:103234364/csab:103238835 | 3 | 3 | 103238335/103234364/103238835 | 0 |  |
| csab04380 | Osteoclast differentiation | 4/175 | 101/5900 | 0.3517153 | 0.7302892 | 103241278/103243277/103223257/103224900 | IL1B/ITGB3/NCF4/PIK3R3 | csab:103241278/csab:103243277/csab:103223257/csab:103224900 | 4 | 1 | 103243277 | 3 | 103241278/103223257/103224900 |
| csab04670 | Leukocyte transendothelial migration | 4/175 | 101/5900 | 0.3517153 | 0.7302892 | 103230197/103241901/103223257/103224900 | RASSF5/CLDN1/NCF4/PIK3R3 | csab:103230197/csab:103241901/csab:103223257/csab:103224900 | 4 | 1 | 103241901 | 3 | 103230197/103223257/103224900 |
| csab04932 | Non-alcoholic fatty liver disease | 5/175 | 132/5900 | 0.3540796 | 0.7302892 | 103226390/103241278/103224900/103214788/103223920 | IL6/IL1B/PIK3R3/IRS2/IL6R | csab:103226390/csab:103241278/csab:103224900/csab:103214788/csab:103223920 | 5 | 2 | 103226390/103214788 | 3 | 103241278/103224900/103223920 |
| csab04612 | Antigen processing and presentation | 2/175 | 43/5900 | 0.3662365 | 0.7433089 | 103223677/103221664 | HSPA6/LOC103221664 | csab:103223677/csab:103221664 | 2 | 0 |  | 2 | 103223677/103221664 |
| csab05321 | Inflammatory bowel disease | 2/175 | 43/5900 | 0.3662365 | 0.7433089 | 103226390/103241278 | IL6/IL1B | csab:103226390/csab:103241278 | 2 | 1 | 103226390 | 1 | 103241278 |
| csab05212 | Pancreatic cancer | 3/175 | 73/5900 | 0.3688389 | 0.7433089 | 103224652/103246489/103224900 | GADD45A/CCND1/PIK3R3 | csab:103224652/csab:103246489/csab:103224900 | 3 | 2 | 103224652/103246489 | 1 | 103224900 |
| csab00480 | Glutathione metabolism | 2/175 | 44/5900 | 0.3769633 | 0.7539266 | 103220822/103218702 | ODC1/MGST1 | csab:103220822/csab:103218702 | 2 | 2 | 103220822/103218702 | 0 |  |
| csab00603 | Glycosphingolipid biosynthesis - globo and isoglobo series | 1/175 | 16/5900 | 0.3826873 | 0.7576869 | 103234996 | FUT1 | csab:103234996 | 1 | 1 | 103234996 | 0 |  |
| csab04974 | Protein digestion and absorption | 3/175 | 75/5900 | 0.3850982 | 0.7576869 | 103214795/103242775/103238196 | COL4A1/COL20A1/SLC38A2 | csab:103214795/csab:103242775/csab:103238196 | 3 | 2 | 103214795/103238196 | 1 | 103242775 |
| csab04152 | AMPK signaling pathway | 4/175 | 107/5900 | 0.3924931 | 0.7576869 | 103246489/103224900/103223243/103214788 | CCND1/PIK3R3/HMGCR/IRS2 | csab:103246489/csab:103224900/csab:103223243/csab:103214788 | 4 | 3 | 103246489/103223243/103214788 | 1 | 103224900 |
| csab04916 | Melanogenesis | 3/175 | 76/5900 | 0.3931936 | 0.7576869 | 103222156/103223483/103224207 | EDN1/WNT7B/WNT2B | csab:103222156/csab:103223483/csab:103224207 | 3 | 1 | 103222156 | 2 | 103223483/103224207 |
| csab05414 | Dilated cardiomyopathy | 3/175 | 76/5900 | 0.3931936 | 0.7576869 | 103243277/103217090/103219220 | ITGB3/CACNB4/TPM2 | csab:103243277/csab:103217090/csab:103219220 | 3 | 2 | 103243277/103217090 | 1 | 103219220 |
| csab00532 | Glycosaminoglycan biosynthesis - chondroitin sulfate / dermatan sulfate | 1/175 | 17/5900 | 0.4010473 | 0.7641070 | 103215414 | CSGALNACT1 | csab:103215414 | 1 | 1 | 103215414 | 0 |  |
| csab04015 | Rap1 signaling pathway | 6/175 | 172/5900 | 0.4023139 | 0.7641070 | 103243277/103230197/103225530/103246479/103223064/103224900 | ITGB3/RASSF5/EPHA2/FGFR3/ADORA2A/PIK3R3 | csab:103243277/csab:103230197/csab:103225530/csab:103246479/csab:103223064/csab:103224900 | 6 | 2 | 103243277/103225530 | 4 | 103230197/103246479/103223064/103224900 |
| csab05150 | Staphylococcus aureus infection | 2/175 | 47/5900 | 0.4086753 | 0.7663513 | 103221732/103233571 | LOC103221732/CFD | csab:103221732/csab:103233571 | 2 | 0 |  | 2 | 103221732/103233571 |
| csab04750 | Inflammatory mediator regulation of TRP channels | 3/175 | 78/5900 | 0.4093013 | 0.7663513 | 103241278/103235528/103224900 | IL1B/PLA2G4C/PIK3R3 | csab:103241278/csab:103235528/csab:103224900 | 3 | 0 |  | 3 | 103241278/103235528/103224900 |
| csab04923 | Regulation of lipolysis in adipocytes | 2/175 | 48/5900 | 0.4190743 | 0.7690801 | 103224900/103214788 | PIK3R3/IRS2 | csab:103224900/csab:103214788 | 2 | 1 | 103214788 | 1 | 103224900 |
| csab04110 | Cell cycle | 4/175 | 111/5900 | 0.4194982 | 0.7690801 | 103224652/103246489/103237430/103237062 | GADD45A/CCND1/MYC/E2F5 | csab:103224652/csab:103246489/csab:103237430/csab:103237062 | 4 | 4 | 103224652/103246489/103237430/103237062 | 0 |  |
| csab04910 | Insulin signaling pathway | 4/175 | 111/5900 | 0.4194982 | 0.7690801 | 103238870/103224900/103214788/103228406 | SOCS2/PIK3R3/IRS2/CBLB | csab:103238870/csab:103224900/csab:103214788/csab:103228406 | 4 | 3 | 103238870/103214788/103228406 | 1 | 103224900 |
| csab04660 | T cell receptor signaling pathway | 3/175 | 80/5900 | 0.4252803 | 0.7727623 | 103218491/103224900/103228406 | PTPN6/PIK3R3/CBLB | csab:103218491/csab:103224900/csab:103228406 | 3 | 1 | 103228406 | 2 | 103218491/103224900 |
| csab04370 | VEGF signaling pathway | 2/175 | 49/5900 | 0.4293806 | 0.7727623 | 103235528/103224900 | PLA2G4C/PIK3R3 | csab:103235528/csab:103224900 | 2 | 0 |  | 2 | 103235528/103224900 |
| csab04512 | ECM-receptor interaction | 3/175 | 81/5900 | 0.4332152 | 0.7727623 | 103243277/103220724/103214795 | ITGB3/SDC1/COL4A1 | csab:103243277/csab:103220724/csab:103214795 | 3 | 2 | 103243277/103214795 | 1 | 103220724 |
| csab04659 | Th17 cell differentiation | 3/175 | 81/5900 | 0.4332152 | 0.7727623 | 103226390/103241278/103223920 | IL6/IL1B/IL6R | csab:103226390/csab:103241278/csab:103223920 | 3 | 1 | 103226390 | 2 | 103241278/103223920 |
| csab05320 | Autoimmune thyroid disease | 1/175 | 19/5900 | 0.4361540 | 0.7727829 | 103237469 | TG | csab:103237469 | 1 | 0 |  | 1 | 103237469 |
| csab04927 | Cortisol synthesis and secretion | 2/175 | 50/5900 | 0.4395906 | 0.7736794 | 103238335/103234364 | NR4A1/LDLR | csab:103238335/csab:103234364 | 2 | 2 | 103238335/103234364 | 0 |  |
| csab04144 | Endocytosis | 7/175 | 214/5900 | 0.4510930 | 0.7846542 | 103246479/103234364/103224044/103233004/103223677/103228406/103239806 | FGFR3/LDLR/PIP5K1A/EHD1/HSPA6/CBLB/DNM1 | csab:103246479/csab:103234364/csab:103224044/csab:103233004/csab:103223677/csab:103228406/csab:103239806 | 7 | 3 | 103234364/103224044/103228406 | 4 | 103246479/103233004/103223677/103239806 |
| csab05165 | Human papillomavirus infection | 9/175 | 281/5900 | 0.4558012 | 0.7846542 | 103243277/103248148/103246489/103223483/103230198/103224207/103214795/103224900/103239621 | ITGB3/DLG2/CCND1/WNT7B/IKBKE/WNT2B/COL4A1/PIK3R3/NOTCH1 | csab:103243277/csab:103248148/csab:103246489/csab:103223483/csab:103230198/csab:103224207/csab:103214795/csab:103224900/csab:103239621 | 9 | 4 | 103243277/103248148/103246489/103214795 | 5 | 103223483/103230198/103224207/103224900/103239621 |
| csab04340 | Hedgehog signaling pathway | 2/175 | 52/5900 | 0.4597082 | 0.7846542 | 103219690/103246489 | GAS1/CCND1 | csab:103219690/csab:103246489 | 2 | 2 | 103219690/103246489 | 0 |  |
| csab04976 | Bile secretion | 2/175 | 52/5900 | 0.4597082 | 0.7846542 | 103234364/103223243 | LDLR/HMGCR | csab:103234364/csab:103223243 | 2 | 2 | 103234364/103223243 | 0 |  |
| csab05166 | Human T-cell leukemia virus 1 infection | 6/175 | 183/5900 | 0.4608978 | 0.7846542 | 103226390/103246489/103236305/103237430/103216128/103224900 | IL6/CCND1/IL15/MYC/EGR2/PIK3R3 | csab:103226390/csab:103246489/csab:103236305/csab:103237430/csab:103216128/csab:103224900 | 6 | 5 | 103226390/103246489/103236305/103237430/103216128 | 1 | 103224900 |
| csab05215 | Prostate cancer | 3/175 | 85/5900 | 0.4645366 | 0.7846542 | 103215685/103246489/103224900 | PLAT/CCND1/PIK3R3 | csab:103215685/csab:103246489/csab:103224900 | 3 | 1 | 103246489 | 2 | 103215685/103224900 |
| csab00010 | Glycolysis / Gluconeogenesis | 2/175 | 53/5900 | 0.4696100 | 0.7846542 | 103242625/103218486 | ALDOC/ENO2 | csab:103242625/csab:103218486 | 2 | 0 |  | 2 | 103242625/103218486 |
| csab04970 | Salivary secretion | 2/175 | 53/5900 | 0.4696100 | 0.7846542 | 103238835/103244467 | ATP2B1/SLC12A2 | csab:103238835/csab:103244467 | 2 | 2 | 103238835/103244467 | 0 |  |
| csab04142 | Lysosome | 4/175 | 119/5900 | 0.4725758 | 0.7846542 | 103242313/103245241/103229330/103243148 | CD68/CTSH/NPC2/ARSG | csab:103242313/csab:103245241/csab:103229330/csab:103243148 | 4 | 1 | 103229330 | 3 | 103242313/103245241/103243148 |
| csab04664 | Fc epsilon RI signaling pathway | 2/175 | 54/5900 | 0.4794036 | 0.7855440 | 103235528/103224900 | PLA2G4C/PIK3R3 | csab:103235528/csab:103224900 | 2 | 0 |  | 2 | 103235528/103224900 |
| csab04724 | Glutamatergic synapse | 3/175 | 87/5900 | 0.4799171 | 0.7855440 | 103235528/103238195/103238196 | PLA2G4C/SLC38A1/SLC38A2 | csab:103235528/csab:103238195/csab:103238196 | 3 | 2 | 103238195/103238196 | 1 | 103235528 |
| csab00515 | Mannose type O-glycan biosynthesis | 1/175 | 22/5900 | 0.4850139 | 0.7855440 | 103242981 | MGAT5B | csab:103242981 | 1 | 0 |  | 1 | 103242981 |
| csab00790 | Folate biosynthesis | 1/175 | 22/5900 | 0.4850139 | 0.7855440 | 103237812 | LOC103237812 | csab:103237812 | 1 | 1 | 103237812 | 0 |  |
| csab05203 | Viral carcinogenesis | 5/175 | 155/5900 | 0.4894151 | 0.7878389 | 103215465/103246489/103218917/103216128/103224900 | EGR3/CCND1/TRAF1/EGR2/PIK3R3 | csab:103215465/csab:103246489/csab:103218917/csab:103216128/csab:103224900 | 5 | 3 | 103215465/103246489/103216128 | 2 | 103218917/103224900 |
| csab00630 | Glyoxylate and dicarboxylate metabolism | 1/175 | 23/5900 | 0.5003461 | 0.8005538 | 103240824 | ACAT2 | csab:103240824 | 1 | 1 | 103240824 | 0 |  |
| csab04662 | B cell receptor signaling pathway | 2/175 | 57/5900 | 0.5081132 | 0.8032449 | 103218491/103224900 | PTPN6/PIK3R3 | csab:103218491/csab:103224900 | 2 | 0 |  | 2 | 103218491/103224900 |
| csab04920 | Adipocytokine signaling pathway | 2/175 | 57/5900 | 0.5081132 | 0.8032449 | 103225587/103214788 | TNFRSF1B/IRS2 | csab:103225587/csab:103214788 | 2 | 1 | 103214788 | 1 | 103225587 |
| csab00030 | Pentose phosphate pathway | 1/175 | 24/5900 | 0.5152243 | 0.8096382 | 103242625 | ALDOC | csab:103242625 | 1 | 0 |  | 1 | 103242625 |
| csab01524 | Platinum drug resistance | 2/175 | 61/5900 | 0.5447626 | 0.8410370 | 103218702/103224900 | MGST1/PIK3R3 | csab:103218702/csab:103224900 | 2 | 1 | 103218702 | 1 | 103224900 |
| csab04260 | Cardiac muscle contraction | 2/175 | 61/5900 | 0.5447626 | 0.8410370 | 103217090/103219220 | CACNB4/TPM2 | csab:103217090/csab:103219220 | 2 | 1 | 103217090 | 1 | 103219220 |
| csab05211 | Renal cell carcinoma | 2/175 | 61/5900 | 0.5447626 | 0.8410370 | 103220342/103224900 | EPAS1/PIK3R3 | csab:103220342/csab:103224900 | 2 | 0 |  | 2 | 103220342/103224900 |
| csab04062 | Chemokine signaling pathway | 4/175 | 133/5900 | 0.5606150 | 0.8556891 | 103235789/103242198/103224900/103242710 | CXCL1/CXCL16/PIK3R3/CCL2 | csab:103235798/csab:103242198/csab:103224900/csab:103242710 | 4 | 0 |  | 4 | 103235789/103242198/103224900/103242710 |
| csab04972 | Pancreatic secretion | 2/175 | 63/5900 | 0.5623663 | 0.8556891 | 103238835/103244467 | ATP2B1/SLC12A2 | csab:103238835/csab:103244467 | 2 | 2 | 103238835/103244467 | 0 |  |
| csab00410 | beta-Alanine metabolism | 1/175 | 28/5900 | 0.5704594 | 0.8556891 | 103227954 | ABAT | csab:103227954 | 1 | 0 |  | 1 | 103227954 |
| csab00640 | Propanoate metabolism | 1/175 | 28/5900 | 0.5704594 | 0.8556891 | 103227954 | ABAT | csab:103227954 | 1 | 0 |  | 1 | 103227954 |
| csab04710 | Circadian rhythm | 1/175 | 28/5900 | 0.5704594 | 0.8556891 | 103227939 | BHLHE40 | csab:103227939 | 1 | 1 | 103227939 | 0 |  |
| csab04935 | Growth hormone synthesis, secretion and action | 3/175 | 102/5900 | 0.5878726 | 0.8672564 | 103238870/103224900/103214788 | SOCS2/PIK3R3/IRS2 | csab:103238870/csab:103224900/csab:103214788 | 3 | 2 | 103238870/103214788 | 1 | 103224900 |
| csab04915 | Estrogen signaling pathway | 3/175 | 103/5900 | 0.5945490 | 0.8672564 | 103244654/103224900/103223677 | HBEGF/PIK3R3/HSPA6 | csab:103244654/csab:103224900/csab:103223677 | 3 | 1 | 103244654 | 2 | 103224900/103223677 |
| csab04520 | Adherens junction | 2/175 | 67/5900 | 0.5961036 | 0.8672564 | 103240591/103218491 | WASF1/PTPN6 | csab:103240591/csab:103218491 | 2 | 1 | 103240591 | 1 | 103218491 |
| csab04650 | Natural killer cell mediated cytotoxicity | 2/175 | 67/5900 | 0.5961036 | 0.8672564 | 103218491/103224900 | PTPN6/PIK3R3 | csab:103218491/csab:103224900 | 2 | 0 |  | 2 | 103218491/103224900 |
| csab05020 | Prion disease | 6/175 | 211/5900 | 0.6012873 | 0.8672564 | 103226390/103241278/103223257/103224900/103239621/103223677 | IL6/IL1B/NCF4/PIK3R3/NOTCH1/HSPA6 | csab:103226390/csab:103241278/csab:103223257/csab:103224900/csab:103239621/csab:103223677 | 6 | 1 | 103226390 | 5 | 103241278/103223257/103224900/103239621/103223677 |
| csab05412 | Arrhythmogenic right ventricular cardiomyopathy | 2/175 | 68/5900 | 0.6042299 | 0.8672564 | 103243277/103217090 | ITGB3/CACNB4 | csab:103243277/csab:103217090 | 2 | 2 | 103243277/103217090 | 0 |  |
| csab04217 | Necroptosis | 3/175 | 105/5900 | 0.6076920 | 0.8672564 | 103241278/103235528/103240296 | IL1B/PLA2G4C/TNFAIP3 | csab:103241278/csab:103235528/csab:103240296 | 3 | 1 | 103240296 | 2 | 103241278/103235528 |
| csab04611 | Platelet activation | 3/175 | 105/5900 | 0.6076920 | 0.8672564 | 103243277/103235528/103224900 | ITGB3/PLA2G4C/PIK3R3 | csab:103243277/csab:103235528/csab:103224900 | 3 | 1 | 103243277 | 2 | 103235528/103224900 |
| csab04973 | Carbohydrate digestion and absorption | 1/175 | 31/5900 | 0.6077365 | 0.8672564 | 103224900 | PIK3R3 | csab:103224900 | 1 | 0 |  | 1 | 103224900 |
| csab04514 | Cell adhesion molecules | 3/175 | 106/5900 | 0.6141574 | 0.8707961 | 103220287/103220724/103241901 | NRXN1/SDC1/CLDN1 | csab:103220287/csab:103220724/csab:103241901 | 3 | 2 | 103220287/103241901 | 1 | 103220724 |
| csab00051 | Fructose and mannose metabolism | 1/175 | 32/5900 | 0.6194329 | 0.8707961 | 103242625 | ALDOC | csab:103242625 | 1 | 0 |  | 1 | 103242625 |
| csab01232 | Nucleotide metabolism | 2/175 | 70/5900 | 0.6201124 | 0.8707961 | 103225017/103240330 | CTPS1/AMPD3 | csab:103225017/csab:103240330 | 2 | 1 | 103225017 | 1 | 103240330 |
| csab04130 | SNARE interactions in vesicular transport | 1/175 | 33/5900 | 0.6307824 | 0.8799232 | 103246629 | STX1A | csab:103246629 | 1 | 0 |  | 1 | 103246629 |
| csab04510 | Focal adhesion | 5/175 | 182/5900 | 0.6332781 | 0.8799232 | 103243277/103246489/103214795/103224900/103224044 | ITGB3/CCND1/COL4A1/PIK3R3/PIP5K1A | csab:103243277/csab:103246489/csab:103214795/csab:103224900/csab:103224044 | 5 | 4 | 103243277/103246489/103214795/103224044 | 1 | 103224900 |
| csab04912 | GnRH signaling pathway | 2/175 | 73/5900 | 0.6430146 | 0.8833599 | 103244654/103235528 | HBEGF/PLA2G4C | csab:103244654/csab:103235528 | 2 | 1 | 103244654 | 1 | 103235528 |
| csab04926 | Relaxin signaling pathway | 3/175 | 111/5900 | 0.6454085 | 0.8833599 | 103222156/103214795/103224900 | EDN1/COL4A1/PIK3R3 | csab:103222156/csab:103214795/csab:103224900 | 3 | 2 | 103222156/103214795 | 1 | 103224900 |
| csab05235 | PD-L1 expression and PD-1 checkpoint pathway in cancer | 2/175 | 74/5900 | 0.6504045 | 0.8833599 | 103218491/103224900 | PTPN6/PIK3R3 | csab:103218491/csab:103224900 | 2 | 0 |  | 2 | 103218491/103224900 |
| csab00620 | Pyruvate metabolism | 1/175 | 35/5900 | 0.6524817 | 0.8833599 | 103240824 | ACAT2 | csab:103240824 | 1 | 1 | 103240824 | 0 |  |
| csab01250 | Biosynthesis of nucleotide sugars | 1/175 | 35/5900 | 0.6524817 | 0.8833599 | 103220112 | GFPT1 | csab:103220112 | 1 | 1 | 103220112 | 0 |  |
| csab04978 | Mineral absorption | 1/175 | 36/5900 | 0.6628510 | 0.8922096 | 103238835 | ATP2B1 | csab:103238835 | 1 | 1 | 103238835 | 0 |  |
| csab04261 | Adrenergic signaling in cardiomyocytes | 3/175 | 115/5900 | 0.6691029 | 0.8922096 | 103217090/103219220/103238835 | CACNB4/TPM2/ATP2B1 | csab:103217090/csab:103219220/csab:103238835 | 3 | 2 | 103217090/103238835 | 1 | 103219220 |
| csab00071 | Fatty acid degradation | 1/175 | 37/5900 | 0.6729125 | 0.8922096 | 103240824 | ACAT2 | csab:103240824 | 1 | 1 | 103240824 | 0 |  |
| csab03050 | Proteasome | 1/175 | 37/5900 | 0.6729125 | 0.8922096 | 103233253 | PSMB10 | csab:103233253 | 1 | 0 |  | 1 | 103233253 |
| csab05168 | Herpes simplex virus 1 infection | 11/175 | 410/5900 | 0.6807114 | 0.8922096 | 103226390/103241278/103243277/103239154/103230198/103224900/103232396/103221664/103219115/103242710/103239156 | IL6/IL1B/ITGB3/OAS1/IKBKE/PIK3R3/NXF3/LOC103221664/ZNF189/CCL2/OAS2 | csab:103226390/csab:103241278/csab:103243277/csab:103239154/csab:103230198/csab:103224900/csab:103232396/csab:103221664/csab:103219115/csab:103242710/csab:103239156 | 11 | 2 | 103226390/103243277 | 9 | 103241278/103239154/103230198/103224900/103232396/103221664/103219115/103242710/103239156 |
| csab00330 | Arginine and proline metabolism | 1/175 | 38/5900 | 0.6826755 | 0.8922096 | 103220822 | ODC1 | csab:103220822 | 1 | 1 | 103220822 | 0 |  |
| csab00514 | Other types of O-glycan biosynthesis | 1/175 | 38/5900 | 0.6826755 | 0.8922096 | 103227904 | GXYLT2 | csab:103227904 | 1 | 1 | 103227904 | 0 |  |
| csab04613 | Neutrophil extracellular trap formation | 3/175 | 118/5900 | 0.6861074 | 0.8922777 | 103243277/103223257/103224900 | ITGB3/NCF4/PIK3R3 | csab:103243277/csab:103223257/csab:103224900 | 3 | 1 | 103243277 | 2 | 103223257/103224900 |
| csab05416 | Viral myocarditis | 1/175 | 40/5900 | 0.7013406 | 0.9031205 | 103246489 | CCND1 | csab:103246489 | 1 | 1 | 103246489 | 0 |  |
| csab01240 | Biosynthesis of cofactors | 3/175 | 121/5900 | 0.7024566 | 0.9031205 | 103218755/103225017/103225584 | BCAT1/CTPS1/DHRS3 | csab:103218755/csab:103225017/csab:103225584 | 3 | 2 | 103218755/103225017 | 1 | 103225584 |
| csab04921 | Oxytocin signaling pathway | 3/175 | 122/5900 | 0.7077613 | 0.9031205 | 103246489/103217090/103235528 | CCND1/CACNB4/PLA2G4C | csab:103246489/csab:103217090/csab:103235528 | 3 | 2 | 103246489/103217090 | 1 | 103235528 |
| csab04024 | cAMP signaling pathway | 4/175 | 161/5900 | 0.7098524 | 0.9031205 | 103222156/103223064/103224900/103238835 | EDN1/ADORA2A/PIK3R3/ATP2B1 | csab:103222156/csab:103223064/csab:103224900/csab:103238835 | 4 | 2 | 103222156/103238835 | 2 | 103223064/103224900 |
| csab04070 | Phosphatidylinositol signaling system | 2/175 | 83/5900 | 0.7115495 | 0.9031205 | 103224900/103224044 | PIK3R3/PIP5K1A | csab:103224900/csab:103224044 | 2 | 1 | 103224044 | 1 | 103224900 |
| csab00520 | Amino sugar and nucleotide sugar metabolism | 1/175 | 44/5900 | 0.7354585 | 0.9242858 | 103220112 | GFPT1 | csab:103220112 | 1 | 1 | 103220112 | 0 |  |
| csab04742 | Taste transduction | 1/175 | 44/5900 | 0.7354585 | 0.9242858 | 103229709 | SCNN1B | csab:103229709 | 1 | 0 |  | 1 | 103229709 |
| csab05152 | Tuberculosis | 3/175 | 129/5900 | 0.7428910 | 0.9242858 | 103226390/103241278/103231401 | IL6/IL1B/ITGAX | csab:103226390/csab:103241278/csab:103231401 | 3 | 1 | 103226390 | 2 | 103241278/103231401 |
| csab00510 | N-Glycan biosynthesis | 1/175 | 46/5900 | 0.7510346 | 0.9242858 | 103242981 | MGAT5B | csab:103242981 | 1 | 0 |  | 1 | 103242981 |
| csab00983 | Drug metabolism - other enzymes | 1/175 | 46/5900 | 0.7510346 | 0.9242858 | 103218702 | MGST1 | csab:103218702 | 1 | 1 | 103218702 | 0 |  |
| csab04730 | Long-term depression | 1/175 | 46/5900 | 0.7510346 | 0.9242858 | 103235528 | PLA2G4C | csab:103235528 | 1 | 0 |  | 1 | 103235528 |
| csab05145 | Toxoplasmosis | 2/175 | 90/5900 | 0.7527327 | 0.9242858 | 103234364/103223677 | LDLR/HSPA6 | csab:103234364/csab:103223677 | 2 | 1 | 103234364 | 1 | 103223677 |
| csab00561 | Glycerolipid metabolism | 1/175 | 47/5900 | 0.7584772 | 0.9270277 | 103224742 | PLPP3 | csab:103224742 | 1 | 0 |  | 1 | 103224742 |
| csab05031 | Amphetamine addiction | 1/175 | 48/5900 | 0.7656985 | 0.9274914 | 103246629 | STX1A | csab:103246629 | 1 | 0 |  | 1 | 103246629 |
| csab04530 | Tight junction | 3/175 | 134/5900 | 0.7658831 | 0.9274914 | 103248148/103246489/103241901 | DLG2/CCND1/CLDN1 | csab:103248148/csab:103246489/csab:103241901 | 3 | 3 | 103248148/103246489/103241901 | 0 |  |
| csab00240 | Pyrimidine metabolism | 1/175 | 49/5900 | 0.7727051 | 0.9277222 | 103225017 | CTPS1 | csab:103225017 | 1 | 1 | 103225017 | 0 |  |
| csab04020 | Calcium signaling pathway | 4/175 | 176/5900 | 0.7731019 | 0.9277222 | 103215153/103246479/103223064/103238835 | GDNF/FGFR3/ADORA2A/ATP2B1 | csab:103215153/csab:103246479/csab:103223064/csab:103238835 | 4 | 2 | 103215153/103238835 | 2 | 103246479/103223064 |
| csab01212 | Fatty acid metabolism | 1/175 | 50/5900 | 0.7795034 | 0.9306290 | 103240824 | ACAT2 | csab:103240824 | 1 | 1 | 103240824 | 0 |  |
| csab04330 | Notch signaling pathway | 1/175 | 51/5900 | 0.7860995 | 0.9306290 | 103239621 | NOTCH1 | csab:103239621 | 1 | 0 |  | 1 | 103239621 |
| csab04924 | Renin secretion | 1/175 | 51/5900 | 0.7860995 | 0.9306290 | 103222156 | EDN1 | csab:103222156 | 1 | 1 | 103222156 | 0 |  |
| csab04622 | RIG-I-like receptor signaling pathway | 1/175 | 52/5900 | 0.7924993 | 0.9330048 | 103230198 | IKBKE | csab:103230198 | 1 | 0 |  | 1 | 103230198 |
| csab03320 | PPAR signaling pathway | 1/175 | 53/5900 | 0.7987087 | 0.9330048 | 103215216 | HMGCS1 | csab:103215216 | 1 | 1 | 103215216 | 0 |  |
| csab04929 | GnRH secretion | 1/175 | 53/5900 | 0.7987087 | 0.9330048 | 103224900 | PIK3R3 | csab:103224900 | 1 | 0 |  | 1 | 103224900 |
| csab04145 | Phagosome | 2/175 | 100/5900 | 0.8027658 | 0.9336131 | 103243277/103223257 | ITGB3/NCF4 | csab:103243277/csab:103223257 | 2 | 1 | 103243277 | 1 | 103223257 |
| csab04071 | Sphingolipid signaling pathway | 2/175 | 102/5900 | 0.8116308 | 0.9369130 | 103224900/103230017 | PIK3R3/DEGS1 | csab:103224900/csab:103230017 | 2 | 1 | 103230017 | 1 | 103224900 |
| csab05208 | Chemical carcinogenesis - reactive oxygen species | 4/175 | 188/5900 | 0.8155357 | 0.9369130 | 103240818/103218702/103224900/103237812 | SOD2/MGST1/PIK3R3/LOC103237812 | csab:103240818/csab:103218702/csab:103224900/csab:103237812 | 4 | 2 | 103218702/103237812 | 2 | 103240818/103224900 |
| csab04918 | Thyroid hormone synthesis | 1/175 | 56/5900 | 0.8162499 | 0.9369130 | 103237469 | TG | csab:103237469 | 1 | 0 |  | 1 | 103237469 |
| csab00310 | Lysine degradation | 1/175 | 57/5900 | 0.8217524 | 0.9391456 | 103240824 | ACAT2 | csab:103240824 | 1 | 1 | 103240824 | 0 |  |
| csab05132 | Salmonella infection | 4/175 | 199/5900 | 0.8485464 | 0.9548781 | 103226390/103241278/103237430/103220717 | IL6/IL1B/MYC/RHOB | csab:103226390/csab:103241278/csab:103237430/csab:103220717 | 4 | 3 | 103226390/103237430/103220717 | 1 | 103241278 |
| csab05170 | Human immunodeficiency virus 1 infection | 3/175 | 157/5900 | 0.8510270 | 0.9548781 | 103225587/103224900/103221664 | TNFRSF1B/PIK3R3/LOC103221664 | csab:103225587/csab:103224900/csab:103221664 | 3 | 0 |  | 3 | 103225587/103224900/103221664 |
| csab05322 | Systemic lupus erythematosus | 1/175 | 63/5900 | 0.8514909 | 0.9548781 | 103221732 | LOC103221732 | csab:103221732 | 1 | 0 |  | 1 | 103221732 |
| csab04371 | Apelin signaling pathway | 2/175 | 113/5900 | 0.8543328 | 0.9548781 | 103215685/103246489 | PLAT/CCND1 | csab:103215685/csab:103246489 | 2 | 1 | 103246489 | 1 | 103215685 |
| csab04360 | Axon guidance | 3/175 | 161/5900 | 0.8627322 | 0.9548781 | 103225530/103246295/103224900 | EPHA2/SLIT2/PIK3R3 | csab:103225530/csab:103246295/csab:103224900 | 3 | 2 | 103225530/103246295 | 1 | 103224900 |
| csab03008 | Ribosome biogenesis in eukaryotes | 1/175 | 66/5900 | 0.8644540 | 0.9548781 | 103232396 | NXF3 | csab:103232396 | 1 | 0 |  | 1 | 103232396 |
| csab04146 | Peroxisome | 1/175 | 66/5900 | 0.8644540 | 0.9548781 | 103240818 | SOD2 | csab:103240818 | 1 | 0 |  | 1 | 103240818 |
| csab04740 | Olfactory transduction | 1/175 | 66/5900 | 0.8644540 | 0.9548781 | 103218440 | ANO2 | csab:103218440 | 1 | 0 |  | 1 | 103218440 |
| csab04658 | Th1 and Th2 cell differentiation | 1/175 | 69/5900 | 0.8762915 | 0.9600046 | 103239621 | NOTCH1 | csab:103239621 | 1 | 0 |  | 1 | 103239621 |
| csab04080 | Neuroactive ligand-receptor interaction | 4/175 | 210/5900 | 0.8764612 | 0.9600046 | 103222156/103239748/103240389/103223064 | EDN1/CALCB/ADM/ADORA2A | csab:103222156/csab:103239748/csab:103240389/csab:103223064 | 4 | 2 | 103222156/103239748 | 2 | 103240389/103223064 |
| csab00562 | Inositol phosphate metabolism | 1/175 | 70/5900 | 0.8800042 | 0.9600046 | 103224044 | PIP5K1A | csab:103224044 | 1 | 1 | 103224044 | 0 |  |
| csab05415 | Diabetic cardiomyopathy | 3/175 | 169/5900 | 0.8837557 | 0.9601296 | 103223257/103224900/103220112 | NCF4/PIK3R3/GFPT1 | csab:103223257/csab:103224900/csab:103220112 | 3 | 1 | 103220112 | 2 | 103223257/103224900 |
| csab05010 | Alzheimer disease | 6/175 | 302/5900 | 0.8916401 | 0.9614866 | 103226390/103241278/103223483/103224207/103224900/103214788 | IL6/IL1B/WNT7B/WNT2B/PIK3R3/IRS2 | csab:103226390/csab:103241278/csab:103223483/csab:103224207/csab:103224900/csab:103214788 | 6 | 2 | 103226390/103214788 | 4 | 103241278/103223483/103224207/103224900 |
| csab04140 | Autophagy - animal | 2/175 | 126/5900 | 0.8933682 | 0.9614866 | 103224900/103214788 | PIK3R3/IRS2 | csab:103224900/csab:103214788 | 2 | 1 | 103214788 | 1 | 103224900 |
| csab04022 | cGMP-PKG signaling pathway | 2/175 | 127/5900 | 0.8959307 | 0.9614866 | 103238835/103214788 | ATP2B1/IRS2 | csab:103238835/csab:103214788 | 2 | 2 | 103238835/103214788 | 0 |  |
| csab04120 | Ubiquitin mediated proteolysis | 2/175 | 130/5900 | 0.9032804 | 0.9654495 | 103227647/103228406 | UBA7/CBLB | csab:103227647/csab:103228406 | 2 | 1 | 103228406 | 1 | 103227647 |
| csab03015 | mRNA surveillance pathway | 1/175 | 79/5900 | 0.9088075 | 0.9664616 | 103232396 | NXF3 | csab:103232396 | 1 | 0 |  | 1 | 103232396 |
| csab04914 | Progesterone-mediated oocyte maturation | 1/175 | 80/5900 | 0.9115490 | 0.9664616 | 103224900 | PIK3R3 | csab:103224900 | 1 | 0 |  | 1 | 103224900 |
| csab04726 | Serotonergic synapse | 1/175 | 84/5900 | 0.9217197 | 0.9733360 | 103235528 | PLA2G4C | csab:103235528 | 1 | 0 |  | 1 | 103235528 |
| csab03013 | Nucleocytoplasmic transport | 1/175 | 89/5900 | 0.9328126 | 0.9734518 | 103232396 | NXF3 | csab:103232396 | 1 | 0 |  | 1 | 103232396 |
| csab04725 | Cholinergic synapse | 1/175 | 89/5900 | 0.9328126 | 0.9734518 | 103224900 | PIK3R3 | csab:103224900 | 1 | 0 |  | 1 | 103224900 |
| csab05022 | Pathways of neurodegeneration - multiple diseases | 7/175 | 373/5900 | 0.9332755 | 0.9734518 | 103226390/103241278/103223483/103225587/103224207/103246629/103227647 | IL6/IL1B/WNT7B/TNFRSF1B/WNT2B/STX1A/UBA7 | csab:103226390/csab:103241278/csab:103223483/csab:103225587/csab:103224207/csab:103246629/csab:103227647 | 7 | 1 | 103226390 | 6 | 103241278/103223483/103225587/103224207/103246629/103227647 |
| csab04141 | Protein processing in endoplasmic reticulum | 2/175 | 147/5900 | 0.9365786 | 0.9734518 | 103233048/103223677 | HERPUD1/HSPA6 | csab:103233048/csab:103223677 | 2 | 1 | 103233048 | 1 | 103223677 |
| csab04928 | Parathyroid hormone synthesis, secretion and action | 1/175 | 93/5900 | 0.9405496 | 0.9735816 | 103244654 | HBEGF | csab:103244654 | 1 | 1 | 103244654 | 0 |  |
| csab03010 | Ribosome | 1/175 | 95/5900 | 0.9440792 | 0.9735816 | 103221259 | RPL22L1 | csab:103221259 | 1 | 1 | 103221259 | 0 |  |
| csab04722 | Neurotrophin signaling pathway | 1/175 | 102/5900 | 0.9548697 | 0.9786122 | 103224900 | PIK3R3 | csab:103224900 | 1 | 0 |  | 1 | 103224900 |
| csab03040 | Spliceosome | 1/175 | 104/5900 | 0.9575531 | 0.9786122 | 103223677 | HSPA6 | csab:103223677 | 1 | 0 |  | 1 | 103223677 |
| csab00230 | Purine metabolism | 1/175 | 106/5900 | 0.9600779 | 0.9786122 | 103240330 | AMPD3 | csab:103240330 | 1 | 0 |  | 1 | 103240330 |
| csab05017 | Spinocerebellar ataxia | 1/175 | 114/5900 | 0.9687685 | 0.9817860 | 103224900 | PIK3R3 | csab:103224900 | 1 | 0 |  | 1 | 103224900 |
| csab05034 | Alcoholism | 1/175 | 116/5900 | 0.9706293 | 0.9817860 | 103223064 | ADORA2A | csab:103223064 | 1 | 0 |  | 1 | 103223064 |
| csab05016 | Huntington disease | 3/175 | 242/5900 | 0.9775851 | 0.9850476 | 103240818/103246306/103246629 | SOD2/TGM2/STX1A | csab:103240818/csab:103246306/csab:103246629 | 3 | 0 |  | 3 | 103240818/103246306/103246629 |
| csab05012 | Parkinson disease | 2/175 | 198/5900 | 0.9831203 | 0.9868584 | 103223064/103227647 | ADORA2A/UBA7 | csab:103223064/csab:103227647 | 2 | 0 |  | 2 | 103223064/103227647 |
| csab05014 | Amyotrophic lateral sclerosis | 2/175 | 284/5900 | 0.9984205 | 0.9984205 | 103225587/103232396 | TNFRSF1B/NXF3 | csab:103225587/csab:103232396 | 2 | 0 |  | 2 | 103225587/103232396 |
